# Influence of soil amendment of different concentrations of amino acid water-soluble fertilizer on physiological characteristics, yield and quality of “Hangjiao No.2” Chili Pepper

**DOI:** 10.7717/peerj.12472

**Published:** 2021-11-29

**Authors:** Emily Patience Bakpa, Jianming Xie, Jing Zhang, Kangning Han, Yufeng Ma, Tiandong Liu

**Affiliations:** College of Horticulture, Gansu Agricultural University, Lanzhou, Gansu, China

**Keywords:** Amino acid water-soluble fertilizer, “Hangjiao No.2” pepper, Fertilization, Fruit quality

## Abstract

Amino acids are well known as natural stimulators of plant growth and are widely used to promote crop yield and quality. Several studies have been conducted to investigate the effects of amino acid (s) as a foliar spray on a variety of plant species. However, the effects of soil amendment of different concentrations of amino acid water-soluble fertilizer on the physiological characteristics, yield, and quality of pepper remain unclear. Following this, three experimental sets of amino acid water-soluble fertilizer in the ratio 1.8: 2.7: 3.6 kg including control (CK) were conducted in Lintao county, Gansu province. The treatments were applied through furrow method at 6 weeks after planting. The results showed that physiological characteristics of the pepper plants, such as chlorophyll a (1.35 mg g^−1^), and b (0.67 mg g^−1^), total chlorophyll (2.02 mg g^−1^), carotenoid (0.63 mg g^−1^), ETR (26.25 µmol m^−2^s^−1^), Fv/Fm (0.75), Qp (0.92) contents of the leaves were increased by the 1.8 kg treatment while NPQ (71.37%) and root activity (2185.52 µg g^−1^ h^−1^) were improved by the 3.6 kg treatment compared to the control. Fertilization with 2.7 kg of amino acid water-soluble fertilizer also had a significant influence on fruit length (25.50 cm), and yield of pepper (37.92 t ha^−1^) while fruit diameter (24.51 mm), firmness (5.30 kg cm^−2^), fresh (48.10 g) and dry (4.71 g) weights were higher in the 1.8 kg treatment compared to the control. The lowest rate of fertilizer (1.8 kg) applied again resulted in a significant increase in soluble protein (79.79%), capsaicin (5.80 mg g^−1^), dihydrocapsaicin (1.08 mg g^−1^), vitamin C (72.33%) and the essential and non-essential amino acid contents of the pepper which ranged from (235.15 to 11.16 µg g^−1^) and (1,605.10 to 16.63 µg g^−1^) respectively, while soluble sugar (9.02%) was enhanced by 3.6 kg treatment compared to the control. The findings suggest that soil amendment with low concentration of amino acid water-soluble fertilizer (1.8 kg) could be successfully used to improve the physiological characteristics and fruit quality of peppers in vegetable production.

## Introduction

Pepper (*Capsicum annuum* L.) is a common condiment and active vegetable ingredient in many cuisines throughout the world. The crop belongs to the Solanoideae subgroup of the Solanaceae family, and there are about 2000 to 3000 species cultivated in different regions of the world (Schmid & Hunziker, 2001; Knapp et al., 2004). Pepper is one of China’s most popular vegetable, and its diverse nutritional profile is critical for dietary diversification and national food security. Chili peppers are the world’s most abundant source of capsaicinoids, ranking first among vegetable crops (Bosland & Votava, 2012).

The globe is today confronted with the dual challenges of feeding an increasing population while simultaneously safeguarding the environment and creating sustainable energy sources (Canellas et al., 2015). To meet these demands, food demand is estimated to increase 2–5 fold by 2030, while food production is expected to expand by 60% in the next decades (St. Clair & Lynch, 2010). Shuqin & Fang (2018) reported that agricultural intensification in China was fueled by non-renewable energy inputs (synthetic fertilizers) in the previous century. Although synthetic fertilizers are beneficial inputs for increasing crop productivity, higher doses of these chemicals to crops are associated with lower agricultural produce quality and productivity, nitrogen dioxide pollution of groundwater, high nitrous oxide emission, soil acidification and salinization, all of which are detrimental to the ecological and sustainable environment over time and other health related issues (Lockwood, Daniel & Wilson, 2004; Guo et al., 2010; Roelcke, Schleef & Richter, 2004). The use of these chemical fertilizers in crop production is increasing at an alarming rate, and show no indications of slowing down (Flores-Sanchez et al., 2011). Furthermore, it is commonly known that increasing agricultural activities would worsen the negative effects of global climate change, and increasing food security uncertainty (El Bilali et al., 2019). In addition, the current trend of vegetables consumption among people in both developed and developing world are now focused on organic products and high nutrient elements (Van de Vijver & van Vliet, 2012; Apaolaza et al., 2018). In an effort to limit the use of synthetic fertilizers, increase marketable yield, improve crop quality and reduce the environmental and ecological repercussions of these synthetic fertilizers, the Chinese Ministry of Agriculture implemented a zero-growth synthetic fertilizer policy (Ministry of Agriculture, 2015). Therefore, broadening the knowledge on soil fertilization with amino acid water-soluble fertilizer may become an option to maximize effective pepper production as well as quality while minimizing the high utilization of chemical fertilizers in pepper production.

Amino acid water-soluble fertilizers are organic and cost-effective source of naturally generated water-soluble nitrogen that is easy to handle, free of phytotoxicity, salt build-up, and soil leaching. As a result, market growth is being driven by increased demand for effective water-soluble fertilizers as well as increased need for nitrogen-rich fertilizers. Furthermore, rising demand for food security for an ever-growing population, limited agricultural land availability, increased crop loss due to nutrient deficiency, increased government investments, and increased awareness about soil profiles are expected to drive up demand for amino acid water-soluble fertilizers (Popko et al., 2018). Because amino acid fertilizers are totally soluble in water, they can be mixed into the injector, sprayer, or irrigation system as needed. Nearly all of the nitrogen in amino acid water-soluble fertilizers is water soluble, making it readily available to plants. The use of amino acid water-soluble fertilizers provides stress tolerance, chelating action, soil and flora equilibrium, and effective protein synthesis (Souri, 2016). Amino acid fertilizers are type of modern fertilizers that are made from different acids and are involved in reducing ammonium influx as well as transporter transcripts in root tissues (Shekari & Javanmardi, 2017). Previous studies on amino acids showed that, when compared to conventional fertilizers, amino acid fertilizers improve growth and photosynthetic properties, which can contribute to the generation and transport of photosynthets to various crop parts (Davies, 2010; Radkowski & Radkowska, 2018; Masclaux-Daubresse et al., 2010). Foliar application of amino acids improves nitrogen uptake efficiency from the soil and reduces nitrogen leaching, according to studies on the absorption and assimilation of various types of nitrogen, such as nitrates and ammonium (Souri, 2016; Shafeek et al., 2018; Liu et al., 2008). Amino acids act as an active catalyst in metabolic processes and influence physiological aspects of metabolism (Shafeek et al., 2012). In comparison to chemical fertilizers, amino acid fertilizers improve soil physicochemical properties and contain a wide spectrum of bioactive chemicals that can improve vegetable crop nutrient usage efficiency, improving yield and quality while cutting pesticide costs and conserving the environment (Wang et al., 2014). According to (Wahba, Motawe & Ibrahim, 2015), amino acid fertilizers have previously shown to be the most important in plant nutrition for increasing yields and quality, as well as shortening the productive cycle and producing better dry material. Several studies have shown that foliar spraying of amino acid combinations has a good effect on plants, such as increased output in *Solanum lycopersicum* L. (Koukounaras, Tsouvaltzis & Siomos, 2013)and accumulation of dry matter and chlorophyll (chl) in soybean plants (Abd El-Aal, 2018).

China has over 1.3 million hectares of pepper cultivation land, making it the world’s second-largest planting area after India (FAO, 2017). In Lintao County of Gansu province of China, several varieties of pepper are cultivated by farmers. However, “Hangjiao No.2” chili pepper used in this study, is the common variety grown. It is a first-generation hybrid pepper developed by Tianshui Lvpeng Agricultural Technology Co., Ltd. in 2005. Significant traits such as rapid growth, tolerance to low temperature, light, and high humidity as well as resistance to stress and viral diseases makes it superior to other varieties such as “Hangjiao No.1” and Tianshui yangjiaojiao. Furthermore, the pepper is suited to both open field and protected cultivation and is well-known for its high-yield spicy seasonal seeds. The fruit is long sheep horn shaped, with flesh thickness of 0.22–0.30 cm. It has excellent commercial characteristics, a powerful spicy flavour, a delicate texture, and a pleasant taste. The crop is usually harvested and consumed when it attains full green maturity. Its cultivation, on the other hand, often necessitates fertilizer inputs in order to sustain significant growth, productivity, and quality. Pepper farmers rely heavily on chemical chemicals (nitrogenous fertilizers) to enhance production while maintaining quality in order to meet increasing demand. Crop productivity necessitates the application of particular nutrients, which can be either above or below ground (Shaheen et al., 2010). Organic fertilizer application is a well-known agricultural practice for improving soil physicochemical qualities and biological processes (Diacono & Montemurro, 2010). Soil fertilization improves the texture and structure of the soil, allowing nutrients to be absorbed and encouraging root growth (Baldi & Toselli, 2013). According to Keller (2005), when nutrient solutions for crops are appropriately integrated with soil management strategies, they can also help alter environmental variables and function as supplement to compensate for deficient soil.

Despite the importance of amino acid fertilizers in crop production, there is little information on the effect of soil amendment of amino acid water-soluble fertilizer in pepper production as most studies have used single or mixtures of amino acids with foliar spray as the most common application method. Due to the importance of pepper in China’s economy and the role of amino acid base fertilizers in crops, the current study aims to determine the influence of different concentrations of amino acid water-soluble fertilizer on the physiological indexes, yield, fruit characteristics, and internal fruit qualities of “Hangjiao No.2” pepper with the prospect of reducing the high quantity of chemical fertilizers (particularly nitrogenous source of fertilizers) used in crop production.

## Materials & Methods

The field experiment was conducted in Lintao County, Gansu province, China (Coordinates; 350 22′38″N 1030 51′41″E) from June to October 2020. Annual rainfall ranges between 30 and 600 mm with a steadily decreasing south-east to north-west gradient, with summer accounting for 58.8% of total precipitation. The evapotranspiration is 2710 mm per year with a 120 day per year frost-free period. The annual average temperature is 7.7 °C and the climate varies between summer and winter with an average temperature difference of 28 °C. The average annual wind speed is 2.8 ms^−1^ and the soil type in the area is clay-loam with flat terrain. Prior to the start of the study, soil samples were collected from the field and analyzed in the lab. The physicochemical properties of the soil such as nitrogen (34.23 mg kg^−1^), potassium (62.11 mg kg^−1^), phosphorus (54.23 mg kg^−1^), pH (6.86), Zinc (15.57 mg kg^−1^), Na (151.21 mg kg^−1^), and Fe (38.95 mg kg^−1^) were determined using standard laboratory procedure as described by Arnon (1949) before planting. The amino acid water-soluble fertilizer (organic fertilizer) used in this study was purchased from Gansu Lvnengriuqi Co., Ltd. The fertilizer is a brown liquid containing fourteen (14) amino acids and other trace elements (Table 1). The “Hangjiao No.2” pepper seeds used in this study were obtained from Tianshui Shenzhou Lvpeng Agricultural Technology Co., Ltd.

**Table 1:** Components of amino acid water-soluble fertilizer.

**Components**	**Concentration (g L**^−1^)
Aspartic acid	10.62
Threonine	4.75
Serine	17.38
Glutamic acid	9.91
Glycine	19.39
Valine	7.39
Methionine	0.65
Isoleucine	4.67
Leucine	3.87
Tyrosine	1.15
Phenylalanine	6.81
Lysine	2.61
Arginine	9.06
Proline	13.92
Boron	3.65
Zinc	3.65
Iron	8.58
Manganese	2.45

### Experimental design and treatment applications

The experiment in this study was arranged in a randomized complete block design (RCBD) and the treatments were replicated 3 times. The planting area for each plot constituted 3 ridges and each ridge was 8.8 m long with 1 m between ridges. The treatments were: CK (control as conventional fertilizer used by farmers), T1 (1.8 kg of amino acid water-soluble fertilizer), T2 (2.7 kg of amino acid water-soluble fertilizer), and T3 (3.6 kg of amino acid water-soluble fertilizer). The seeds were soaked in 55 °C water for 15 min with constant stirring and then at 25 °C for 8 h before being spread on damp towel and placed in an electronic climate box at 28 °C and 70% relative humidity for 4 days to enhance uniform germination. The germinated seeds with about three mm buds were selected and then seeded into seedling trays containing soil at the experimental laboratory of the College of Horticulture, Gansu Agricultural University at 25 °C and 68% relative humidity. Watering of seedlings was done twice during the germination period. The seedlings were then transported at the 6-true leaf stage to the experimental field prior to transplanting. Potassium sulphate compound fertilizer (187 kg ha^−1^) was applied as the base to the entire plot 2 weeks before planting, while nitro sulfur-based nitrogen fertilizer (3 kg) containing total nitrogen (26%), nitrate nitrogen (8%), sulfur (11%) and zinc (0.02%) was applied as the control according to the customary quantity of fertilizer used by farmers. At 6 weeks after transplanting, each ratio (T1–T3) of the amino acid water-soluble fertilizer was diluted with 50 litres of water, thoroughly mixed and equally distributed per furrow with a watering can. The treatments were applied 3 times at 2-week interval. The furrow irrigation method was adopted to supply water to the crops and there was no pesticide application. Nine plants were randomly selected from each treatment for the measurement of physiological characteristics. Fruits from each treatment were also selected for postharvest qualities, with some samples being dried and powdered and others being frozen for biochemical analysis.

### Pepper leaf analysis

#### The concentration of photosynthetic pigments in the leaves

Nine (9) leaves of fully developed functional pepper plants were randomly selected after treatment applications. A 0.5 diameter puncture was used to obtain (0.1 g) sample leaves from each treatment for photosynthetic pigment evaluation. The samples obtained were then submerged in 10 mL of 80% acetone containing 20 mL of graduated test tubes with a stopper and left in the dark for 48 h with 8 h of oscillation interval. Acetone (80%) was zeroed when the leaves turned white and the OD values of the extracted solutions were measured at 663 nm, 645 nm, and 440 nm with a UV-1780 spectrometer (Shimadzu, Japan), following the method described by Arnon, (1949).

#### Measurements of chlorophyll fluorescence parameters of the leaves

Nine (9) pepper leaves were randomly selected from each treatment and dark-adapted for 30 min after harvest. The electron transport rate (ETR), the maximal quantum yield of PSII photochemistry (Fv/Fm), non-photochemical quenching coefficient (NPQ), photochemical quenching coefficient (qP), and quantum efficiency of PSII photochemistry Y (II) were measured with an imaging-PAM chlorophyll fluorometer (Walz Effeltrich Germany) using a procedure described by (Lu et al., 2019) after 30 min of dark-adaption.

### Root activity

The Triphenyltetrazoliun chloride (TTC) procedure was used to estimate root activity (Ruff et al., 1987). The pepper root from each treatment was cut into parts. The Triphenyltetrazoliun chloride (0.1 mL) standard solution was added to 0.5 g of each sample in a 10 mL volumetric flask and well shaken. The volume on the scale was then fixed using ethyl acetate. 0.25, 0.50, 1.00, 1.5, and two mL of the solutions were placed in a 10 mL volumetric flask, respectively. Acetic acid ethyl chloride was added to each volume to reach the mark. The Colorimetric series containing water-insoluble triphenylmethyl hydrazone (TTF) was measured at 485 nm to draw the standard curve. The curve was checked to calculate the reduction amount of Triphenyltetrazoliun chloride.

### Determination of pepper yield and fruits physiochemical characteristics

The pepper fruits were harvested when fully grown and recorded as total fruits per area. The average weight of fruits obtained from each plot was converted to (t ha^−1^). Fruits from the second branches of each treatment were randomly selected for physicochemical characteristics. Nine (9) fruits were also selected from each treatment for fruit length and diameter determination using a measuring tape and digital calliper, respectively. The fresh and dry weights of pepper fruits were determined with a digital measuring scale. Pepper firmness was measured using a portable penetrometer, and the results obtained were expressed in (kg cm^−2^).

### Quantification of capsaicin and dihydrocapsaicin concentrations in pepper fruits

The capsaicin and dihydrocapsaicin contents were evaluated using High Performance Liquid Chromatography (HPLC). Eighteen (18) pepper fruits were randomly selected after harvest at the green mature stage, washed with distilled water, wiped with a clean tissue, and weighed with an electronic balance. The weighed samples were then dried at 55 °C in an electronic oven until a constant weight was obtained. The oven-dried pepper fruits were ground into powder for the evaluation. The ground samples (1.0 g) were extracted with methanol-tetrahydrofuran (1:1, v: v) using the SB25-12D temperature-controlled ultrasonic machine (Ningbo Scientz Biotech company, China) at 60 °C for 3 times. The solution obtained was filtered through a 0.22 µm Millipore membrane size following the procedure described by (Barbero et al., 2008). Capsaicin and dihydrocapsaicin (Sigma, USA, 98%) were weighed and dissolved accurately in (1.0 mg mL^−1^) methanol and 0, 20, 40, 60, 80, and 100 µg mL^−1^ were also prepared with the mixed standard solutions. The High Performance Liquid Chromatography (HPLC) system used in this analysis consisted of a Shimadzu LC-20A HPLC (Kyoto, Japan) with a ZORBAX Eclipse Plus C18 column (250 mm ×4.6 mm ×5.0 µm). A Shimadzu UV–VIS 1700 spectrophotometer was used to measure the absorbance at 280 nm. Aqueous solution of methanol-1% acetic acid was used as the mobile phase at 1.0 mL min^−1^, 30 °C column temperature, and 5 µL injection volume as the flow rate. Capsaicin and dihydrocapsaicin contents calculated from the standard curve were expressed in (mg g^−1^) on a dry weight.

### Vitamin C, soluble sugar and protein content of pepper fruits

Vitamin C was measured by the 2,6-dichloroindophenol staining method (Arya, Mahajan & Jain, 2000). The Anthrone sulfuric acid assay method was used to estimate soluble sugar content (Cataldo et al., 1975). Coomassie brilliant blue technique was used to estimate the soluble protein content (Sedmak & Grossberg, 1977).

### Essential and non-essential amino acids determination

Liquid chromatography tandem mass spectrometry was used to determine the individual amino acids. Dried pepper from each treatment was selected at random and extracted with hydrochloric acid (0.1%) by ultrasonic. The samples were then injected into small bottles and the amino acid contents determined directly after centrifugal separation treatment. The individual amino acids were separated through liquid chromatography on a reversed-phase Xterra MS C18 (50 mm ×2.1 mm ×2.5 µm), 0.1% formic acid solution and acetonitrile were taken as mobile phases, and the results were expressed as µg g^−1^ (Zhang et al., 2017).

### Statistical analysis

The data collected were subjected to analysis of variance (one-way ANOVA), and Tukey’s Honest Significant Difference at 5% was used to determine significant differences in the means using the IBM SPSS software package (version 20.0). Microsoft Excel 2019 was used to draw graphs with standard error bars.

**Figure 1:**
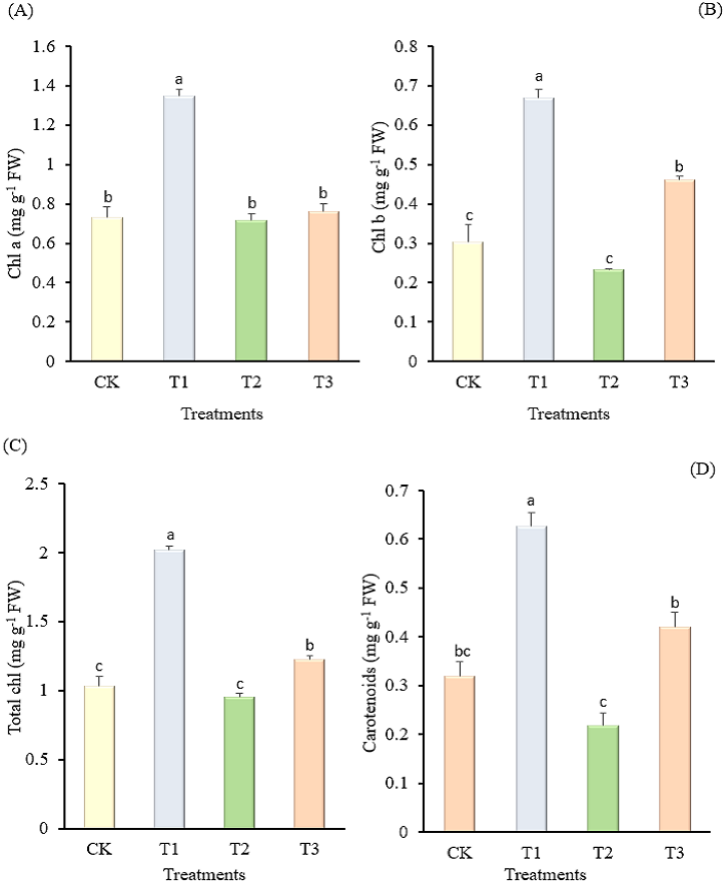
Effect of different concentrations of amino acid water-soluble fertilizer on chl a (A) and b (B), total chl (C) and carotenoid contents (D) of “Hangjiao No.2” pepper leaves. Mean values with different alphabets differ significantly at (*p* < 0.05) by Tukey’s test.

## Results

Figure 1 shows that when the pepper plants were treated with amino acid water-soluble fertilizer, the chl content increased at the lowest concentration of fertilizer applied. Additionally, the 1.8 kg amino acid water-soluble fertilizer treatment significantly increased chl a (1.35 mg g^−1^), whereas the 2.7 kg amino acid water-soluble fertilizer treatment significantly decreased chl a (0.72 mg g^−1^), which was non-significant to the control (0.73 mg g^−1^) and the 3.6 kg amino acid water-soluble fertilizer treatment (0.76 mg g^−1^) at *p* > 0.05. A similar trend was observed for chl b, with the chl b content of the leaves increasing as the fertilizer concentration (1.8 kg) was decreased. More precisely, the 1.8 kg amino acid water-soluble fertilizer treatment had much more chl b (0.67 mg g^−1^) compared to 2.7 kg amino acid water-soluble fertilizer treatment (0.23 mg g^−1^), which was similar to the control (0.30 mg g^−1^). The findings in (Fig. 1C) indicated significant differences in total chl among the treatments tested. The total chl content of “Hangjiao No.2” pepper was enhanced by 1.8 kg amino acid water-soluble fertilizer (2.02 mg g^−1^), whereas 2.7 kg treatment and the control decreased the total chl content by (0.95 mg g^−1^) and (1.03 mg g^−1^), respectively. The 1.8 kg amino acid water-soluble fertilizer treatment on the other hand, significantly increased the carotenoid content (0.63 mg g^−1^) when compared to the 2.7 kg amino acid water-soluble fertilizer treatment and the control, both of which had the lowest carotenoid content values of 0.22 mg g^−1^ and 0.32 mg g^−1^, respectively.

**Figure 2:**
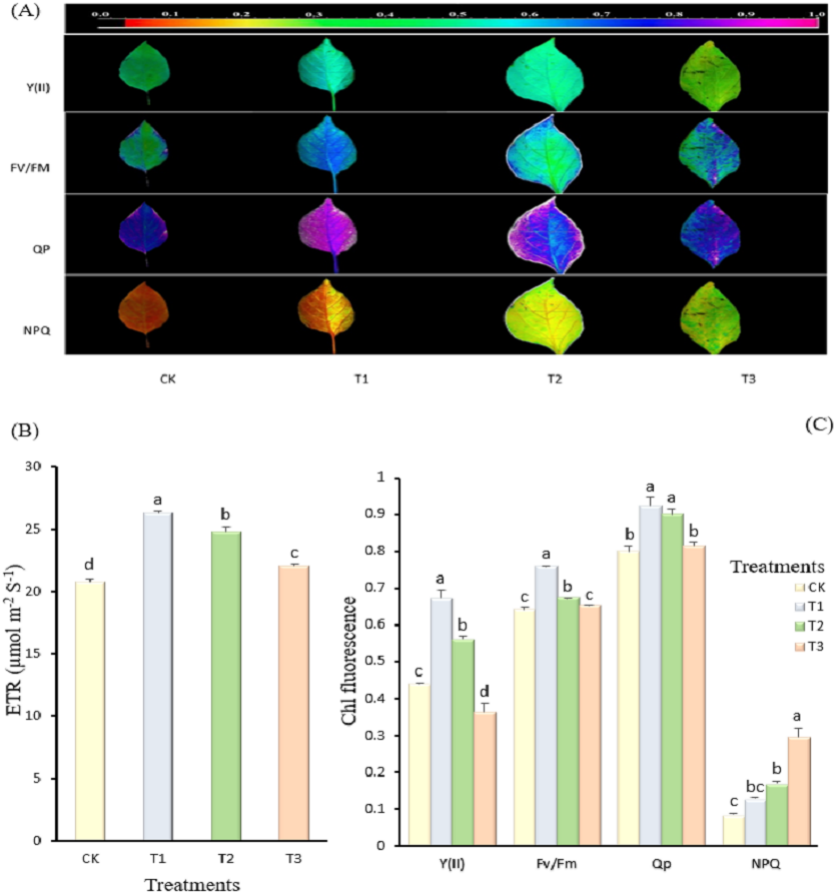
(A–C) Effect of different concentrations of amino acid water-soluble fertilizer on electron transport rate (ETR) (B), quantum efficiency of PSII photochemistry Y(II), maximal quantum yield of PSII photochemistry (Fv/Fm), photochemical quenching coefficient (Qp) and non-photochemical quenching coefficient (NPQ) (C) of “Hangjiao No.2” pepper leaves. Mean values with different alphabets differ significantly at (*p* < 0.05) by Tukey’s test. The coloured bar at the top of the leaf images (A) represents the chlorophyll fluorescence parameters range black (0) to purple (1.0) and how they mapped to the colour palette.

**Figure 3:**
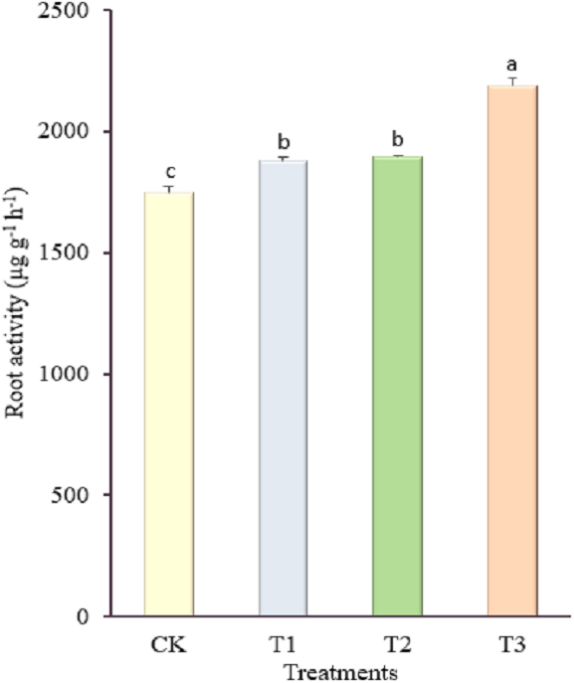
Effect of different concentrations of amino acid water-soluble fertilizer on root activity of “Hangjiao No.2” pepper plants. Mean values with different alphabets differ significantly at (*p* < 0.05) by Tukey’s test.

The electron transport rates (ETR) of the pepper leaves were affected significantly by the different treatments applied, as shown in (Fig. 2F). Pepper plants treated with 1.8 kg amino acid water-soluble fertilizer had the maximum uptake of electron transport rate (26.25 µmol m^−2^ s^−1^), whereas the control had the lowest electron transport rate (21.00 µmol m^−2^ s^−1^). The lowest concentration of amino acid water-soluble fertilizer (1.8 kg) applied significantly increased the quantum efficiency of PSII photochemistry (0.67), whereas the highest concentration of fertilizer (3.6 kg) applied significantly decreased the quantum efficiency of PSII photochemistry (0.36). Furthermore, irrespective of the treatments, the control and 3.6 kg amino acid water-soluble fertilizer treatments reduced the maximum quantum yield of PSII photochemistry by 0.64 and 0.65 respectively, whereas the 1.8 kg amino acid water-soluble fertilizer treatment increased the maximum quantum yield of PSII photochemistry (0.76). There was no significant difference in photochemical quenching coefficient between the 1.8 kg and 2.7 kg amino acid water-soluble fertilizer treatments, but these treatments had the highest photochemical quenching coefficient of 0.92 and 0.90 respectively, while the lowest photochemical quenching coefficient was observed between the control (0.80) and the 3.6 kg amino acid water-soluble fertilizer treatment (0.81). The non-photochemical quenching coefficient of the pepper leaves differed significantly among the treatments. The non-photochemical quenching coefficient obtained improved when the concentration of amino acid water-soluble fertilizer was increased. As 3.6 kg of amino acid water-soluble fertilizer was applied to the pepper leaves, the non-photochemical quenching coefficient content increased by 71.37% when compared to the control. The application of amino acid water-soluble fertilizer at different concentrations improved root activity of pepper plants to varying degrees than the control, as shown in (Fig. 3). In comparison to the control (1,747.18 µg g^−1^ h^−1^), increasing the dosage of amino acid water-soluble fertilizer applied to 3.6 kg resulted in a significant increase in root activity (2,185.52 µg g^−1^ h^−1^) of the pepper plant (*p* <  0.00).

### Fruit length, fruit diameter, fresh fruit weight and fruit dry weight

There were significant differences in the treatments used for fruit qualities measured as shown in (Table 2). The amino acid water-soluble fertilizer treatments had a significant effect on fruit length when compared to the control. Pepper plants treated with 2.7 kg amino acid water-soluble fertilizer had the longest fruit length (25.50 cm) among the amino acid water-soluble fertilizer treatments, while the control had the shortest fruit length (17.20 cm). The results for fruit diameter measurement showed that the treatments had a significant effect on fruit diameter (Table 2). Additionally, decreasing the concentration of the fertilizer had a greater effect on the average diameter of “Hangjiao No.2” pepper fruits. Pepper plants treated with 1.8 kg of amino acid water-soluble fertilizer had a wider fruit diameter (24.51 mm) compared to those fertilized with 3.6 kg of amino acid water-soluble fertilizer (18.22 mm). A similar trend was observed for pepper fresh weight, with the average weight of “Hangjiao No.2” pepper increased when the concentration of amino acid water-soluble fertilizer applied decreased. The 1.8 kg treatment resulted in the heaviest fresh fruit weight (48.10 g), while the 3.6 kg treatment resulted in the least fresh weight (41.22 g). In comparison to 3.6 kg of amino acid water-soluble fertilizer treatment, application of 1.8 kg of amino acid water-soluble fertilizer on “Hangjiao No.2” pepper plants improved fruit fresh weight and diameter. The dry weight of the pepper varied depending on the treatments used. The average dry weight of the pepper (4.71 g) was significantly higher in plants treated with 1.8 kg amino acid water-soluble fertilizer (*p* <  0.00), while the control had the lowest dry weight (2.45 g).

**Table 2:** Effect of different concentrations of amino acid water-soluble fertilizer on some fruit characteristics of “Hangjiao No.2” pepper. Means with different alphabets within the same column differ significantly from each other (*p* < 0.05). Values are means ± standard error.

**Treatment**	**Fruit length** **(cm)**	**Fruit diameter** **(mm)**	**Fresh weight** **(g)**	**Dry weight** **(g)**
CK	17.20 ± 0.50c	19.54 ± 0.18c	42.92 ± 0.27c	2.45 ± 0.17c
T1	21.13 ± 0.72b	24.51 ± 0.16a	48.10 ± 0.34a	4.71 ± 0.41a
T2	25.50 ± 0.54a	21.10 ± 0.14b	45.12 ± 0.20b	3.65 ± 0.06b
T3	20.22 ± 0.40b	18.22 ± 0.11d	41.22 ± 0.41d	3.57 ± 0.16b

**Figure 4:**
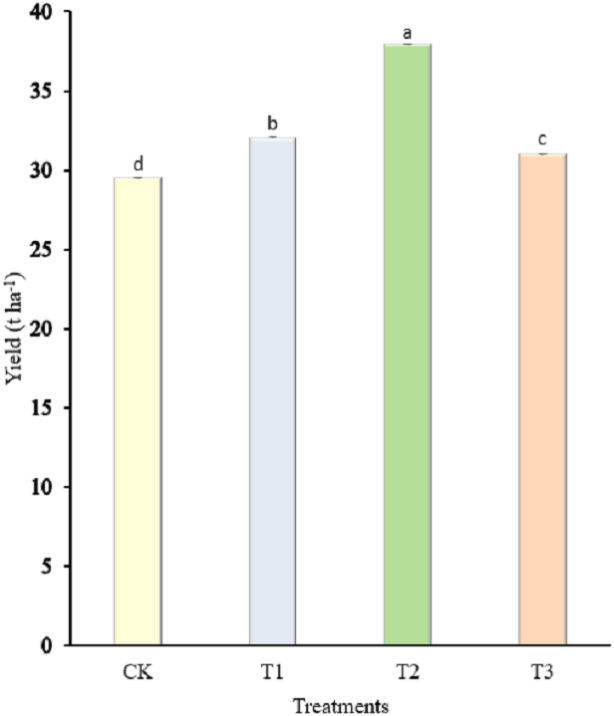
Effect of different concentrations of amino acid water-soluble fertilizer on yield of “Hangjiao No.2” pepper. Mean values with different alphabets differ significantly at (*p* < 0.05) by Tukey’s test.

### Pepper yield

Figure 4 shows the average yield (t ha^−1^) of pepper as affected by the treatments applied at (*p* <  0.05). Comparison of the averages indicated that the highest yield of pepper (37.92 t ha^−1^) obtained was due to the application of 2.7 kg amino acid water-soluble fertilizer, while the lowest yield (29.50 t ha^−1^) was obtained in the control plot.

### Fruit firmness, soluble protein and soluble sugar

The application of amino acid water-soluble fertilizer at different concentrations increased firmness statistically compared to the control, as shown in (Table 3). Plants treated with 1.8 kg of amino acid water-soluble fertilizer recorded firmer “Hangjiao No.2” pepper (5.30 kg cm^−2^) compared to the control (1.60 kg cm^−2^). In contrast, the quantity of soluble sugar in pepper increased significantly as the concentration of fertilizer increased. The soluble sugar content of the pepper increased by 9.02% when 3.6 kg of amino acid water-soluble fertilizer was applied, whereas the soluble sugar content of the fruits decreased by 6.62% and 6.62%, respectively, when 1.8 kg of amino acid water-soluble fertilizer and the control was applied. Furthermore, the soluble protein content in the pepper increased as the concentration of amino acid water-soluble fertilizer used was decreased. In comparison to the control, 1.8 kg of amino acid water-soluble fertilizer increased the soluble protein content by 79.79%.

### Phytochemicals

Table 4 shows some phytochemical analyses of pepper fruits from different treatments applied to “Hangjiao No.2” pepper plants used in this study. Pepper plants treated with 1.8 kg amino acid water-soluble fertilizer had the highest significant capsaicin content of 4.80 mg g^−1^, while the control had the lowest capsaicin content of 2.04 mg g^−1^. The results also indicated that the 1.8 kg amino acid water-soluble fertilizer treatment recorded the highest dihydrocapsaicin (1.08 mg g^−1^) whereas the control and 2.7 kg amino acid water-soluble fertilizer treatment had the lowest dihydrocapsaicin of 0.17 and 0.18 mg g^−1^, respectively. The effects of amino acid water-soluble fertilizer treatments on vitamin C contents were higher compared to the control. In comparison to the control, pepper plants applied with 1.8 kg of amino acid water-soluble fertilizer had the highest significant vitamin C content (72.33%).

**Table 3:** Effect of different concentrations of amino acid water-soluble fertilizer on some postharvest qualities of “Hangjiao No.2”pepper fruits. Means with different alphabets within the same column differ significantly from each other (*p* < 0.05). Values are means ± standard error.

**Treatment**	**Fruit firmness** **(kg/cm)**	**Soluble sugar** **(% FW)**	**Soluble protein** **(mg g** ^−1^ **FW)**
CK	1.60 ± 0.14d	6.62 ± 0.23c	0.92 ± 0.00d
T1	5.30 ± 0.06a	6.62 ± 0.30c	4.56 ± 0.03a
T2	2.92 ± 0.06b	7.67 ± 0.07b	2.39 ± 0.00b
T3	3.16 ± 0.08b	9.02 ± 0.13a	1.22 ± 0.03c

**Table 4:** Effect of different concentrations of amino acid water-soluble fertilizer on Capsaicin, dihydrocapsaicin and vitamin C contents of “Hangjiao No.2” pepper. Means with different alphabets within the same column differ significantly from each other (*p* < 0.05). Values are means ± standard error.

**Treatment**	**Capsaicin** **(mg g** ^−1^ **DW)**	**Dihydrocapsaicin** **(mg g** ^−1^ **DW)**	**Vitamin C** **(mg g** ^−1^ **FW)**
CK	2.04 ± 0.24d	0.17 ± 0.00c	1.58 ± 0.09c
T1	5.80 ± 0.14a	1.08 ± 0.00a	5.71 ± 0.12a
T2	2.72 ± 0.05c	0.18 ± 0.01c	2.63 ± 0.02b
T3	3.75 ± 0.07b	0.25 ± 0.00b	2.63 ± 0.09b

### Essential and non-essential amino acids

Table 5 shows significant changes in 8 essential amino acids of “Hangjiao No.2” pepper fruits treated with different treatments. The application of 1.8 kg amino acid water-soluble fertilizer treatment significantly increased histidine content (235.15 µg g^−1^) of the pepper, but the 3.6 kg amino acid water-soluble fertilizer treatment decreased histidine content (151.69 µg g^−1^). Plants applied with 1.8 kg amino acid water-soluble fertilizer had the highest isoleucine (86.05 µg g^−1^), whereas those fertilized with 3.6 kg amino acid water-soluble fertilizer had the lowest (61.06 µg g^−1^). The elements leucine, phenylalanine, threonine, and valine observed in pepper fruits yielded similar results. Furthermore, pepper fruits applied with 1.8 kg amino acid water-soluble treatment had the highest methionine (11.16 µg g^−1^), while pepper plants applied with 3.6 kg treatment had the lowest methionine (5.01 µg g^−1^). The content of tryptophan of the pepper, on the other hand, showed a similar trend (*p* <  0.00).

**Table 5:** Effect of different concentrations of amino acid water-soluble fertilizer on essential amino acids of “Hangjiao No.2” pepper. Means with different alphabets within the same column differ significantly from each other (*p* < 0.05). Values are means ± standard error.

	**Essential amino acids (µg g** ^−1^ **DW)**							
**Treatment**	**Histidine**	**Isoleucine**	**Leucine**	**Methionine**	**Phenylalanine**	**Threonine**	**Tryptophan**	**Valine**
CK	176.36 ± 0.50c	75.03 ± 0.68c	41.53 ± 0.50c	7.96 ± 0.06b	50.74 ± 0.40c	47.92 ± 0.05c	18.65 ± 0.63b	64.53 ± 0.49c
T1	235.15 ± 0.29a	86.05 ± 0.45a	54.74 ± 0.46a	11.16 ± 0.18a	57.09 ± 0.57a	58.86 ± 0.52a	25.09 ± 0.57a	82.31 ± 0.21a
T2	189.68 ± 0.54b	81.63 ± 0.38b	49.56 ± 0.06b	9.30 ± 0.58b	53.90 ± 0.54b	54.05 ± 0.06b	19.00 ± 0.58b	77.03 ± 0.57b
T3	151.69 ± 0.46d	61.06 ± 0.57d	33.93 ± 0.06d	5.01 ± 0.25c	41.48 ± 0.29d	40.32 ± 0.98d	14.30 ± 0.55c	54.60 ± 0.46d

The results for non-essential amino acids showed significant differences among the treatments applied to the pepper plants, as shown in (Table 6). The application of 1.8 kg amino acid water-soluble fertilizer consistently increased all the non-essential amino acids measured, ranging from 1605.10 µg g^−1^ to 16.63 µg g^−1^, whereas soil amendment with 3.6 kg amino acid water-soluble fertilizer consistently decreased all the non-essential amino acids measured, ranging from 951.24 to 7.60 µg g^−1^ among the treatments applied. However, when compared to the other non-essential amino acids measured, the aspartic acid content in pepper fruits was the highest (T1-1605.10 µg g^−1^, T2-1236.02 µg g^−1^, CK-1104.87 µg g^−1^, and T3-951.24 µg g^−1^), respectively.

**Table 6:** Effect of different concentrations of amino acids water-soluble fertilizer on non-essential amino acids of “Hangjiao No.2” pepper. Means with different alphabets within the same column differ significantly from each other (*p* < 0.05). Values are means ± standard error.

	**Non-essential amino acids (µg g** ^−1^ **DW)**									
**Treatments**	**Alanine**	**Arginine**	**Asparagine**	**Aspartic acid**	**Cysteine**	**Glutamine**	**Glycine**	**Proline**	**Serine**	**Tyrosine**
CK	369.85 ± 0.54c	149.96 ± 0.12b	42.81 ± 0.55c	1104.87 ± 0.61c	11.37 ± 0.45c	80.33 ± 0.71c	12.27 ± 0.29c	134.37 ± 0.51c	164.69 ± 0.52c	152.93 ± 0.11c
T1	450.43 ± 2.95a	191.62 ± 0.36a	72.48 ± 0.28a	1605.10 ± 1.15a	16.63 ± 0.34a	117.51 ± 0.58a	19.13 ± 0.50a	207.60 ± 0.47a	233.06 ± 0.57a	206.84 ± 1.48a
T2	415.55 ± 0.36b	153.63 ± 1.76b	47.31 ± 0.63b	1236.02 ± 1.27b	13.30 ± 0.29b	84.66 ± 0.58b	14.50 ± 0.49b	173.32 ± 0.46b	189.69 ± 0.74b	184.85 ± 0.09b
T3	281.08 ± 0.57d	119.89 ± 0.59c	39.85 ± 0.02d	951.24 ± 0.48d	7.60 ± 0.06d	63.53 ± 0.35d	8.73 ± 0.46d	100.03 ± 1.15d	146.34 ± 0.33d	141.57 ± 0.59d

## Discussion

### Photosynthetic pigments and chlorophyll fluorescence parameters of pepper Leaves

The major photosynthetic pigments, chl a, b, and carotenoids, play a vital role in photosynthesis. Due to the vital role of photosynthesis in plant growth and development, plant physiologists have long considered the concentration of chl in leaves to be a key physiological parameter (Bahari, Pirdashti & Yaghubi, 2013). The application of 1.8 kg amino acid water-soluble fertilizer increased chl a, b, and total chl in this study when compared to the other treatments (Fig. 1). The content of chl can be used as a sensitive indicator of cellular metabolic status; consequently, a decrease in chl suggests tissue toxicity due to ion build-up (Khosravinejad, Heydari & Farboodnia, 2008). A decrease in light absorption and thus the amount of reactive oxygen species synthesized by chloroplasts can be attributed to a decrease in chl content in leaves, according to (Gilmore & Ball, 2000). Chloroplast structural degradation and the instability of pigment protein complexes could be due to the decline in chl a, b, and total chl in the control, 2.7 kg and 3.6 kg treatments, respectively (Jamil et al., 2012).

Carotenoid is a large class of isoprenoid, a natural pigment known for its photoprotective and antioxidant properties, as well as its role as a precursor to phytohormones (Kang et al., 2018; Perera & Yen, 2007). This research also showed that carotenoid level increased when the amount of amino acid water-soluble fertilizer applied was 1.8 kg (Fig. 1D). According to Dong et al. (2007), carotenoid metabolism is proportional to plastid-generated signals, and its functional diversity regulates wide range of physiological activities important for plant growth and development. This implies that the pepper plants’ physiological indicators improved as a result of 1.8 kg amino acid water-soluble fertilizer treatment.

Chl fluorescence is considered a probe of photosynthetic productivity that allows for improvement in crop production strategies (Baker & Rosenqvist, 2020). The state of photosystem II (PSII) and the range at which PSII exploits energy acquired by chl are also reviewed by chl fluorescence (Maxwell & Johnson, 2000). The electron transport rate was improved by 1.8 kg of amino acid water-soluble fertilizer treatment in this study. This suggests that an appropriate application of amino acid water-soluble fertilizer may improve the opening degree of the PSII reaction center and increase their rate of light energy utilization. The maximum quantum yield of PSII photochemistry according to Guo & Li (2000), shows plants’ ability to use light energy for photosynthesis, making it an important source for plant survival and development. The maximum quantum yield of PSII photochemistry is also an important factor in plants’ responses to external environments, and has been used to study the photosynthetic system and predict the growth trend of plants (Ying, Lee & Tollenaar, 2000). Therefore, the highest maximum quantum yield of PSII photochemistry obtained due to the treatment with 1.8 kg of amino acid water-soluble fertilizer may have improved PSII functions and increased chloroplast photochemical activities of the plants (Garousi, 2016). Quantum efficiency of PSII photochemistry is linked with carbon assimilation fixation and has an impact on carbon dioxide assimilation in plants (Souza et al., 2004). According to Gao et al. (2020) and Guan, Møller & Schjoerring (2015), the degree of photoinhibition of crop plants affects the declining amplitude of maximal photochemical efficiency of PSII. This suggests that the lowest maximal quantum yield of PSII photochemistry obtained in both 3.6 kg amino acid water-soluble fertilizer treatment and the control in (Fig. 2G) may have enhanced PSII photoinhibition in pepper. In the present study, 3.6 kg amino acid water-soluble fertilizer treatment resulted in a higher non-photochemical quenching coefficient (0.30) but a lower photochemical quenching coefficient compared to the 1.8 kg amino acid water-soluble fertilizer treatment (Fig. 2G). According to (Wang et al., 2016), an increase in energy dissipation by non-photochemical quenching can lead to a decrease in the maximum relative electron transport rate. Our results suggest that under 3.6 kg amino acid water-soluble fertilizer application, pepper plants may require more effective photoprotection.

### Root activity

The root system serves a variety of activities for crops, such as absorption of moisture from the soil and synthesis of certain hormones that regulate crop growth and development (Dong et al., 2003; Ruf & Brunner, 2003). In addition, it is an important physiological index of crop plants, and is involved in the synthesis of photosynthetic substances (Dalling, Boland & Wilson, 1976). Although there is a general assumption that an improved root system may improve crop performance, the highest root activity obtained in the 3.6 kg amino acid water-soluble fertilizer treatment (Fig. 3), did not influence the characteristics of the pepper plants. According to Thorup-Kristensen & Kirkegaard (2016), excess availability of soil resources and their uptake and use may be limited by the aboveground plant demand for the resources rather than by the root system performance due to factors such as excessive fertilization, high rainfall or suboptimal plant growth. Under these circumstances, modified root growth, though very important for growth and development, may have no effect on production.

### Yield and fruit characteristics of pepper

Nutrient use efficiency improvement in crop production and stabilization of yield by practicing sustainable agriculture is one of the major current issues of food security worldwide (Searchinger et al., 2019). In this context, we evaluated different concentrations of amino acid water-soluble fertilizer on pepper yield. The results showed that soil application of amino acid water-soluble fertilizer was more effective at improving the pepper yield compared to the control. This could be because amino acid water-soluble fertilizers are readily available source of growth elements that aid in the development of protein structures in living tissues. According to Hafez (2012), amino acid fertilizers provide instant-accessible nitrogen that is generally absorbed faster by plant cells than inorganic nitrogen. According to Sarafi et al. (2018), boron can alter total nutrient intake as well as nutrient utilization efficiency, leading to enhanced crop output. Additionally, the highest yield obtained due to application of 2.7 kg amino acid water-soluble fertilizer (Fig. 4), contradicts the findings of Shafeek et al. (2018), who found that foliar application of amino acid mix at a high concentration significantly increased the yield of onion plants.

The application of organic fertilizers to soil should theoretically improve crop growth properties. The results of this study, however, showed that small quantity of amino acid water-soluble fertilizer (organic fertilizer) may improve pepper productivity. Fruit diameter, fresh and dry weight were improved, when 1.8 kg amino acid water-soluble fertilizer was applied compared to the control, except for pepper length, which was increased by the 2.7 kg amino acid water-soluble fertilizer treatment (Table 2). These improvements may be concluded as the ease with which developing components that form proteins in living tissues can be exported (RH & RGhaith, 2014). In addition, the increased dry weight obtained by 1.8 kg amino acid water-soluble fertilizer treatment could be attributed to improved vegetative development, as indicated by improved photosynthesis, which increases the availability of organic nutrients and results in increase dry weight. These findings contradict those of Shafeek et al. (2018), who showed that using higher concentration of amino acid combinations as foliar spray significantly improved most of the onion characteristics compared to lower concentration. These could be due to differences in cultivar type and mode of application.

Fruit firmness is an important textural property of both fruits and vegetables, as well as a potential trait of storage. Fruits and vegetables with firmer texture have a longer storage life because they are more resistant to handling, physical damage and transit, and delay deterioration (Ortiz, Graell & Lara, 2011). The increased accumulation of amino acids, minerals, and protein components present in the amino acid water-soluble fertilizer treatments compared to the control in our current study, may account for the overall improvement in fruit firmness reported (Keutgen & Pawelzik, 2008; Kazem Souri, Sooraki & Moghadamyar, 2017). The firmest fruits obtained due to soil amendment with 1.8 kg amino acid water-soluble fertilizer treatment may help in improving pepper storability.

### Evaluation of internal quality indexes of pepper fruits

#### Concentrations of capsaicin and dihydrocapsaicin

Pepper have long been regarded as a savory spice with a distinct aroma and taste. Pungency, colour, and flavour are all important sensory attributes of hot peppers (Perucka & Oleszek, 2000). Chili pepper and its isolated constituents, such as capsaicinoids, are valued for their medicinal properties, which include antioxidants, anticancer, antibacterial, anti-inflammatory, and immunomodulatory properties (Luo, Peng & Li, 2011). Capsaicin and dihydrocapsaicin make up 69% and 22% of the capsaicinoids in peppers, respectively, and have twice the pungency of nordihydrocapsaicin and homocapsaicin (Kulkarni et al., 2017). The biosynthesis of capsaicinoids is assisted by branched-chain fatty acid and phenylpropanoid pathway, which begins with valine and phenylalanine (Yap et al., 2017). However, the biosynthesis of secondary metabolites (capsaicin and dihydrocapsaicin) in this study was increased due to1.8 kg amino acid water-soluble fertilizer treatment compared to the control (Table 4). This suggests that the highest contents of capsaicin and dihydrocapsaicin detected could be attributed to high contents of valine and phenylalanine recorded by 1.8 kg amino acid water-soluble fertilizer treatment, which are involved in the synthesis of secondary metabolites.

#### Vitamin C

Vitamins are a small group of molecules that are vital for human and animal growth and development (Ou et al., 2017). Pepper is high in vitamins C and E, as well as provitamin A, however its vitamin C content is one of the most noticeable nutritional attributes among vegetable crops (Teodoro et al., 2013). Vitamin C has anticancer properties and enhances collagen synthesis and immune system function (Lutsenko, Cárcamo & Golde, 2002; Frei & Lawson, 2008). Vitamin C contents were significantly influenced by the different concentrations of amino acid water-soluble fertilizer in this study, especially the 1.8 kg amino acid water-soluble fertilizer treatment (5.72 mg g^−1^) as compared to the control (Table 4). This is consistent with the findings of Lei et al. (2019), who found that foliar application of amino acid water-soluble fertilizer at different concentrations improved the vitamin C content of pepper. According to Lee & Kader (2000), agricultural practices, play a vital role in the overall quantity of vitamin C in crops. This suggests that amino acid water-soluble fertilizer may have positive effect on the vitamin C content of pepper.

#### Soluble sugar and protein contents

The accumulation of solutes during fruit growth is an important criterion that can be assessed with better accuracy and is utilized as a reliable harvest index of fruits and vegetables (Sanchez-Sánchez et al., 2010). The highest soluble sugar was 9.02% when the concentration of amino acid water-soluble fertilizer applied was 3.6 kg, which was higher than the control and 1.8 kg treatment (Table 3). Amino acid sequences make up protein, which is the basic important element of living cells (Popko et al., 2018). According to Sameera & Bafeel (2016), nitrogenous fertilizers such as amino acids promote protein concentration in crop plant tissues due to nitrogen’s involvement in protein structure and nucleic acids; thus, the improvement in protein content by amino acid water-soluble fertilizer treatments compared to the control could be due to amino acids being the sole source of nitrogenous fertilizer. Additionally, the increase in soluble protein content due to amino acid water-soluble fertilizer treatments may be attributed to increased soil mineral availability, absorption, translocation, and distribution in plant tissues (Garcia et al., 2011; Yang et al., 2016).

#### Concentration of amino acids

Amino acids are vital for protein synthesis, and their absence in diets can result in protein deficiency disorders (Akram et al., 2014). Amino acids are needed for cellular growth, development, reconstruction, and regeneration as well as the creation of antibodies, blood cells, enzymes, and hormones according to Zou, Ma & Tian (2015). All plants have sufficient nutrients, and improving the concentration of vital nutrients in edible parts of plants while reducing or at least maintaining the concentration of unwanted elements and keeping them within the safe range is vital for human health (Wang, Li & Malhi, 2008). The results in Tables 5 and 6 showed that 1.8 kg amino acid water-soluble fertilizer significantly influenced the contents of both essential and non-essential amino acids among the treatments. Considering the fertilizer’s ecological and economic impacts, it can be concluded that the 1.8 kg amino acid water-soluble fertilizer is the appropriate concentration for obtaining higher content of amino acids of pepper. Furthermore, the decrease in mean values of amino acids observed in the 3.6 kg fertilizer treatment suggests that excessive supply of amino acid water-soluble fertilizer may be detrimental to fruit quality. Aspartic acid was the most abundant, followed by histidine, while methionine was the least, followed by cysteine amino acids found in this study. Fertilization is a simple and effective way to manage and improve the nutritional quality of crops for human consumption. Therefore, application of 1.8 kg water-soluble amino acid fertilizer treatment could help improve the amino acids of pepper.

## Conclusions

The application of amino acid water-soluble fertilizer was aimed to enhance nutrient uptake by plant roots, leading to increased pepper production and quality. In the present study, soil amendment with amino acid water-soluble fertilizer improved the physiological components, yield, fruit characteristics, and quality of “Hangjiao No.2” pepper. The results showed that 2.7 kg treatment may be best for producing higher yield, while soil amendment with 1.8 kg amino acid water-soluble fertilizer may help improve the physiological components, fruit characteristics, and quality of pepper. Based on this study, adding 1.8 kg of amino acid water-soluble fertilizer (organic fertilizer) to the soil 6 weeks after transplanting had the optimal effects. Hence, implementing the 1.8 kg amino acid water-soluble fertilizer treatment and fertilization mechanism by farmers may help improve pepper production as well as quality while minimizing the use of high chemical fertilizers required in pepper production. Studies should be carried out to determine the effects of soil amendment of amino acid water-soluble fertilizer at different concentrations on other vegetable crops.

## Supplemental Information

10.7717/peerj.12472/supp-1 Supplemental Information 1 Raw data of field and laboratory analysis

## Additional Information and Declarations

### Data Availability

The following information was supplied regarding data availability: The raw measurements are provided in the Supplementary Files.
